# Protein S-nitrosation differentially modulates tomato responses to infection by hemi-biotrophic oomycetes of *Phytophthora* spp.

**DOI:** 10.1038/s41438-021-00469-3

**Published:** 2021-02-01

**Authors:** Tereza Jedelská, Michaela Sedlářová, Jan Lochman, Lucie Činčalová, Lenka Luhová, Marek Petřivalský

**Affiliations:** 1 Department of Biochemistry, Palacký University, Šlechtitelů 27, CZ-783 71 Olomouc, Czech Republic; 2 Department of Botany, Faculty of Science, Palacký University, Šlechtitelů 27, CZ-783 71 Olomouc, Czech Republic; 3 Department of Biochemistry, Faculty of Science, Masaryk University, Kamenice 753/5, CZ-625 00 Brno, Czech Republic

**Keywords:** Plant immunity, Plant signalling

## Abstract

Regulation of protein function by reversible S-nitrosation, a post-translational modification based on the attachment of nitroso group to cysteine thiols, has emerged among key mechanisms of NO signalling in plant development and stress responses. S-nitrosoglutathione is regarded as the most abundant low-molecular-weight S-nitrosothiol in plants, where its intracellular concentrations are modulated by S-nitrosoglutathione reductase. We analysed modulations of S-nitrosothiols and protein S-nitrosation mediated by S-nitrosoglutathione reductase in cultivated *Solanum lycopersicum* (susceptible) and wild *Solanum habrochaites* (resistant genotype) up to 96 h post inoculation (hpi) by two hemibiotrophic oomycetes, *Phytophthora infestans* and *Phytophthora parasitica*. S-nitrosoglutathione reductase activity and protein level were decreased by *P. infestans* and *P. parasitica* infection in both genotypes, whereas protein S-nitrosothiols were increased by *P. infestans* infection, particularly at 72 hpi related to pathogen biotrophy–necrotrophy transition. Increased levels of S-nitrosothiols localised in both proximal and distal parts to the infection site, which suggests together with their localisation to vascular bundles a signalling role in systemic responses. S-nitrosation targets in plants infected with *P. infestans* identified by a proteomic analysis include namely antioxidant and defence proteins, together with important proteins of metabolic, regulatory and structural functions. Ascorbate peroxidase S-nitrosation was observed in both genotypes in parallel to increased enzyme activity and protein level during *P. infestans* pathogenesis, namely in the susceptible genotype. These results show important regulatory functions of protein S-nitrosation in concerting molecular mechanisms of plant resistance to hemibiotrophic pathogens.

## Introduction

Nitric oxide (NO) plays key roles in multiple plant developmental processes such as seed dormancy, germination, root development, stomata regulation, flowering and senescence^1^. Within plant interaction with diverse abiotic and biotic stress conditions, NO is involved in interplay with reactive oxygen species (ROS) in the initiation and progression of programmed cell death, hypersensitive reaction and systemic resistance to pathogen infections. NO signalling is mediated through modifications of target proteins through NO binding to metal centres in metalloproteins, S-nitrosation of cysteine thiols or nitration of tyrosine residues^2^. NO signalling via S-nitrosation of regulatory proteins and enzymes proceeds through a reversible transfer of a nitroso group (–NO) to cysteine thiols which results in changes of protein conformations and their biological activities. Multiple experimental studies including proteome-wide scale analysis have explored the nitrosoproteome changes within diverse stress responses and point to the important role of S-nitrosation in plant stress signal transduction^3,4^. In *Arabidopsis thaliana*, cytosolic glyceraldehyde-3-phosphate dehydrogenase was found to be reversibly inhibited by NO, where catalytic Cys155 and Cys159 appear to be the S-nitrosation targets^3,5^. Similarly, methionine adenosyltransferase and Arabidopsis type-II metacaspase AtMC9 are also reversibly inhibited by S-nitrosation^6^. Plasma membrane NADPH-oxidase activity, required for pathogen-induced ROS production and disease resistance in Arabidopsis, is inhibited by S-nitrosation of its Cys890 residue during the hypersensitive reaction^7^. Several proteomic studies have identified components of the ascorbate–glutathione cycle ascorbate peroxidase (APX), glutathione reductase, monodehydroascorbate reductase and dehydroascorbate reductase as targets of S-nitrosation or tyrosine nitration modifications in plants exposed to abiotic stress^4,8–10^. However, if similar regulatory mechanisms of antioxidant enzymes operate also in plants exposed to biotic stress stimuli has not been elucidated yet.

S-nitrosoglutathione reductase (GSNOR, EC 1.1.1.284) is the crucial enzyme in the catabolism of S-nitrosoglutathione (GSNO), a low-molecular weight adduct of glutathione and NO^11^. Due to its ability to metabolise GSNO, GSNOR is indirectly involved in the regulation of protein S-nitrosothiols, which can be produced by a *trans*-nitrosation mechanism involving GSNO and cysteine thiols. GSNOR activity was shown essential for plant development and various stress responses^12–17^. Mutations of the Arabidopsis gene *GSNOR1* have pleiotropic effects on plant development including abnormal stem and trichome branching, flowering disruption and reduced seed production^12–14^. Similarly, GSNOR knockdown in tomato plants resulted in significant alterations of the plant and fruit development^16,17^. Several studies confirmed the involvement of GSNOR-mediated denitrosation in plant interactions with biotic stress factors but with somewhat conflicting conclusions. There is accumulated evidence that the loss or decrease of GSNOR activity results in increased plant susceptibility to pathogens. Lower GSNOR activity and higher levels of S-nitrosothiols compromised basal and non-host resistance in Arabidopsis infected by *Pseudomonas syringae*^18^ or in the resistant cultivar of sunflower infected with oomycete *Plasmopara halstedii*^8^. Salicylic acid (SA)-induced expression of the NPR1-dependent defence gene PR-1 was found suppressed in Arabidopsis GSNOR mutants^19^. Furthermore, the susceptibility of Arabidopsis *par2‐1* mutant line, which contains a point mutation in AtGSNOR1, to bacterial pathogen could be complemented by a constitutive expression of FLAG‐tagged *AtGSNOR1*^20^. In tomato, down-regulation of GSNOR transcript compromised SA-mediated basal resistance, whereas higher GSNOR expression increased pathogen resistance^17^.

On the other hand, an increased resistance against biotrophic oomycete *Peronospora parasitica* in Arabidopsis plants with reduced levels of GSNOR was reported, linked to higher S-nitrosothiol levels, constitutive activation of defence gene *PR-1* and systemic acquired resistance (SAR), whereas an opposite reaction to the pathogen, i.e. increased susceptibility, was observed in plants overexpressing GSNOR^21^. Based on GSNOR localisation into the phloem, GSNO was proposed to function as a NO mobile pool throughout the plant body linked to SAR^22^. GSNOR was differentially modulated in interactions of susceptible and resistant *Lactuca* spp. genotypes with powdery and downy mildew, where a lower S-nitrosothiol level mediated by GSNOR activity was identified as a common characteristic of *Lactuca* spp. reactions to mildew infection^23^. Recently, GSNOR was found down-regulated both on a local and systemic level in three tomatoe (*Solanum* spp.) genotypes infected with a biotrophic pathogen *P. neolycopersici*^24^.

The genus *Phytophthora* represents a group of hemibiotrophic soil-borne oomycetes, whose more than 200 member species cause enormous crop losses as well as forest die-backs worldwide^25^. Actually, no effective control measures for the disease are available and its management relies on the introduction of resistant genotypes. *Phytophthora infestans* (potato or tomato late blight) can cause up to 90% yield losses in some regions of the Czech Republic. The primary infection comes from zoospores released to the wet soil which enter roots or tubers. The disease spreads upwards to the stems and leaves, exhibiting necroses covered by greyish sporulation in humid conditions. In tomato, symptoms include spots on fruits which destroy the yield^26^.

Based on previous studies concerning NO roles in tomato powdery mildew pathogenesis^22,24,27^ and detailed characterisation of tomato GSNOR in vitro^28^, we focused on the pivotal role of tomato GSNOR in interactions with hemibiotrophic oomycetes. Two tomato genotypes cultivated *Solanum lycopersicum* cv. Amateur and wild *Solanum habrochaites*, differing in resistance to plant pathogens, were challenged with *P. infestans* (the most destructive pathogen of Solanaceae plants), and *P. parasitica* (a wide host range pathogen) to study the function of GSNOR in the control of protein S-nitrosation in early tomato responses to pathogen infection.

## Results

### GSNOR activity and protein level are decreased by pathogen infection

**Fig. 1:**
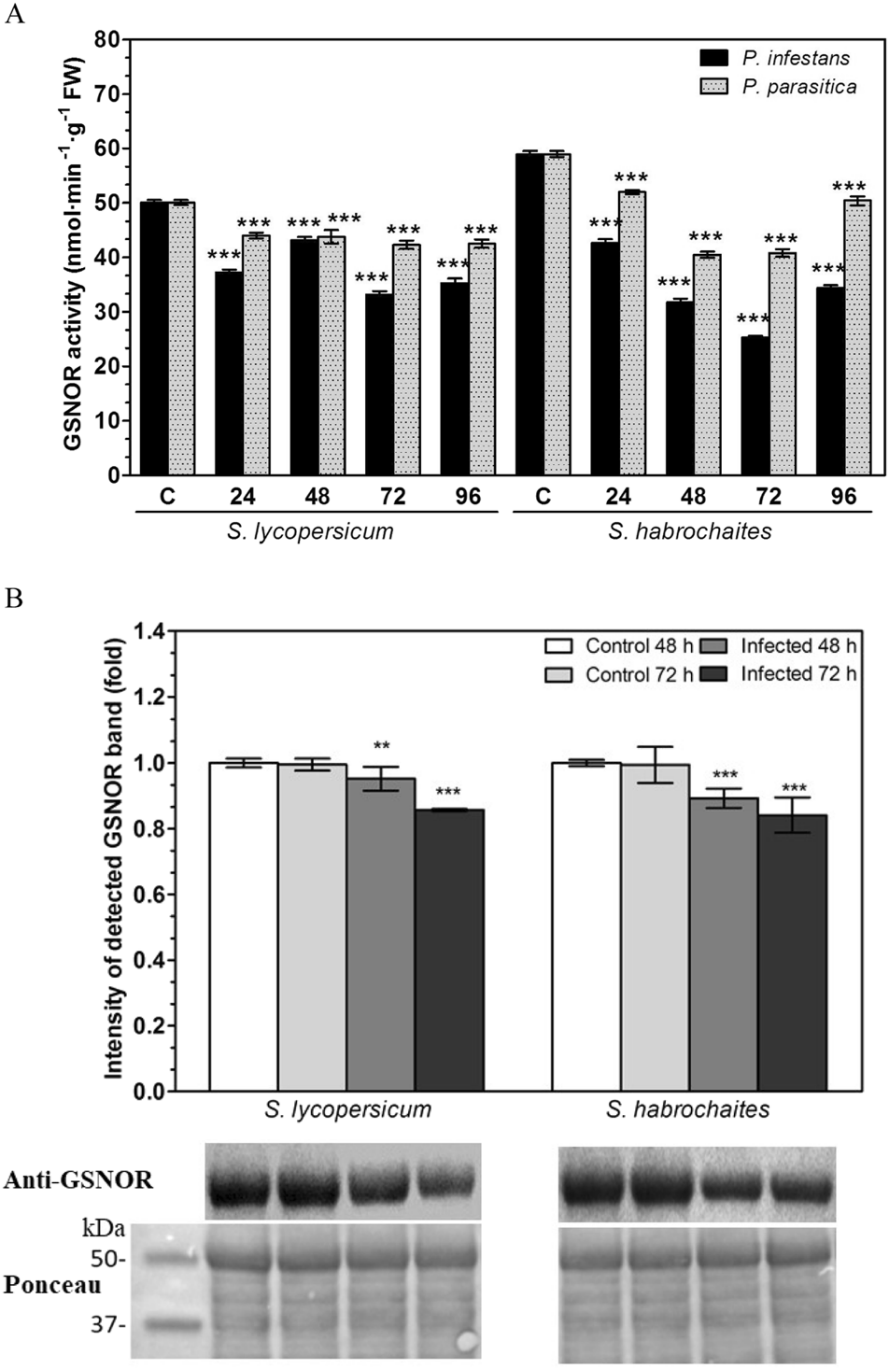
S-nitrosoglutathione reductase (GSNOR) activity and protein levels during the pathogenesis of *P. infestans* and *P. parasitica* on *Solanum* spp. genotypes. **A** GSNOR activity was evaluated in leaf samples collected 0, 24, 48, 72 and 96 hpi of *P. infestans* and *P. parasitica* at 25°C by analysis of NADH absorbance at *λ* = 340 nm. Data represent means ± SD (*n* ≥ 3). Means significantly different from control are denoted by asterisks (ANOVA, *P < 0.05, ***P < 0.001). **B** GSNOR protein levels were quantified in leaf samples (100 μg of protein per lane) inoculated with *P. infestans* 48 and 72 hpi by Western blot analysis using polyclonal rabbit antibody against tomato GSNOR (dilution 1:1000) and goat anti-rabbit IgG peroxidase conjugate (dilution 1:10,000). The intensity of bands was quantified by ImageJ 1.33 software using a non-infected control plant of each genotype as a reference value (=1). Data represent means ± SD (*n* ≥ 3). Significantly different means from the control are denoted by asterisks (ANOVA, **P < 0.01, ***P < 0.001). Reversible Ponceau staining was used as a loading control. The molecular masses of protein standards (kDa) are shown on the *left*

GSNOR activity was determined by spectrophotometric measurement of NADH-dependent reductase activity (Fig. 1A). The basal activity of GSNOR in control non-inoculated plants slightly differed between tomato genotypes, i.e. 50 and 60 nmol min^−1^ g^−1^ FW for *S. lycopersicum* and *S. habrochaites*, respectively (Fig. 1A). Infection by both pathogens caused significantly decreased GSNOR activities recorded from 24 hpi in both genotypes. The most pronounced decrease in GSNOR activity was observed at 72 hpi, i.e. by approximately 35% in *S. lycopersicum* and 60 % in *S. habrochaites*. In general, a decrease in tomato GSNOR activity caused by *P. parasitica* was less pronounced compared to the effect of *P. infestans* infection in all studied time intervals. A relative increase of GSNOR at 96 hpi in comparison to 72 hpi might be related to the biotrophic–necrotrophic transition of the pathogen infection, expected to initiate at 72 hpi. GSNOR protein levels in tomato leaf extracts were quantified by immunoblot analysis using rabbit polyclonal antibody against tomato GSNOR (Fig. 1B). In agreement with the observed decrease in GSNOR activity, the level of GSNOR protein was reduced by pathogenesis in both genotypes (only data for *P. infestans* are shown). GSNOR was localised by immunohistochemical staining with polyclonal antibody previously raised against tomato GSNOR in our laboratory.

**Fig. 2:**
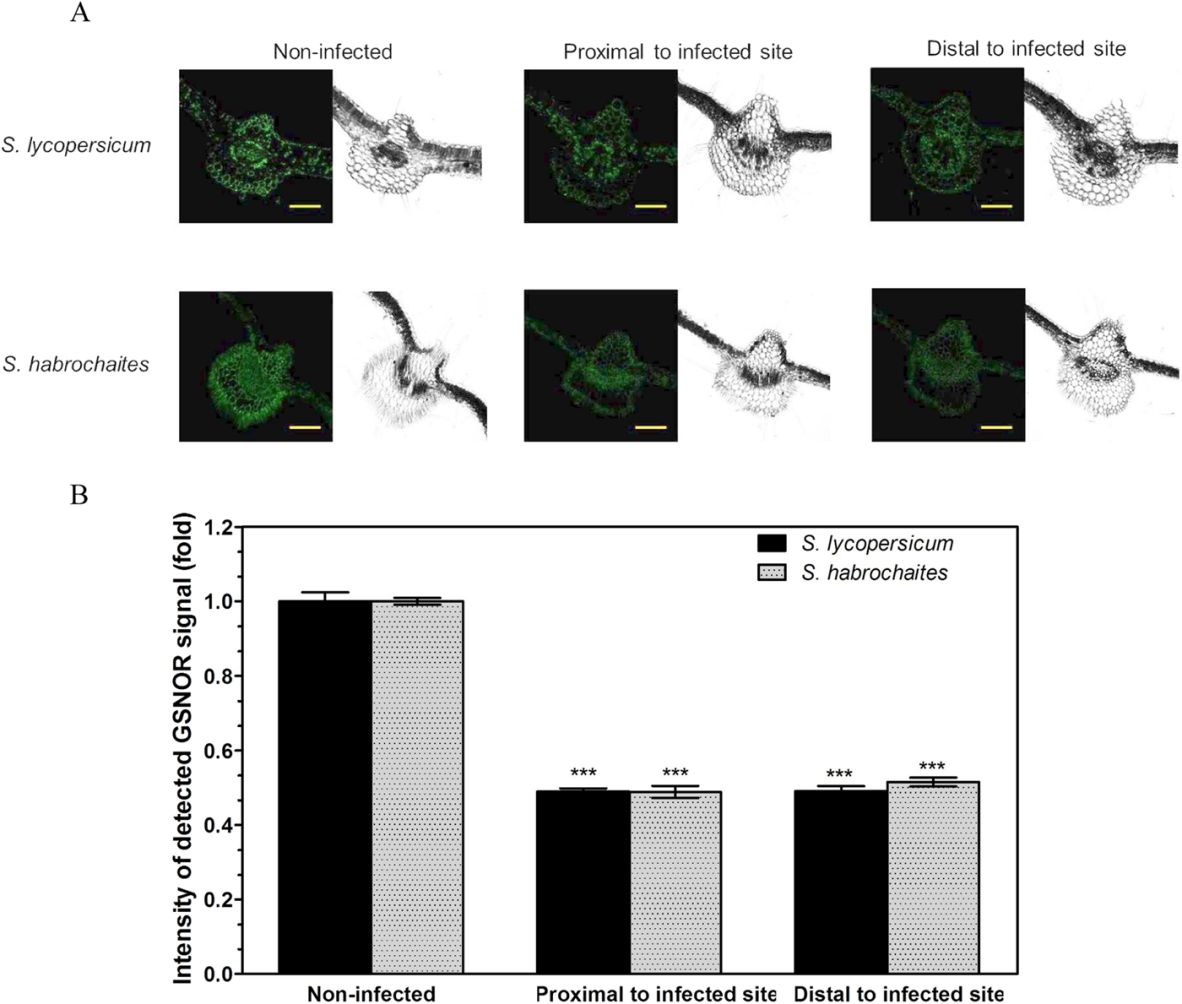
Immunohistochemical detection of GSNOR in leaves of *Solanum* spp. 72 hpi with *P. infestans*. **A** GSNOR was localised in cross-sections of *S. lycopersicum* and *S. habrochaites* infected leaves using rabbit polyclonal antibody against tomato GSNOR and goat anti-rabbit IgG DyLight 488 conjugate. Scale bar = 200 μm. **B** Quantification of fluorescence signal intensity was performed by ImageJ 1.33 software using a non-infected control plant of each genotype as a reference value (=1). Data represent means ± SD (*n* ≥ 3). Means significantly different from the control are denoted by asterisks (ANOVA, ***P < 0.001)

In both tomato genotypes, GSNOR localised to leaflet vascular bundles, parenchyma cells and epidermis, where it was reduced upon infection by *P. infestans* (Fig. 2). No significant differences in the intensity of GSNOR fluorescence signals were found in between leaflet parts proximal and distal to the infection site, respectively. Data for *P. parasitica* are not presented as any changes of measured parameters were found during the studied stages of its pathogenesis.

### Protein S-nitrosothiols are increased in infected tomato plants

**Fig. 3:**
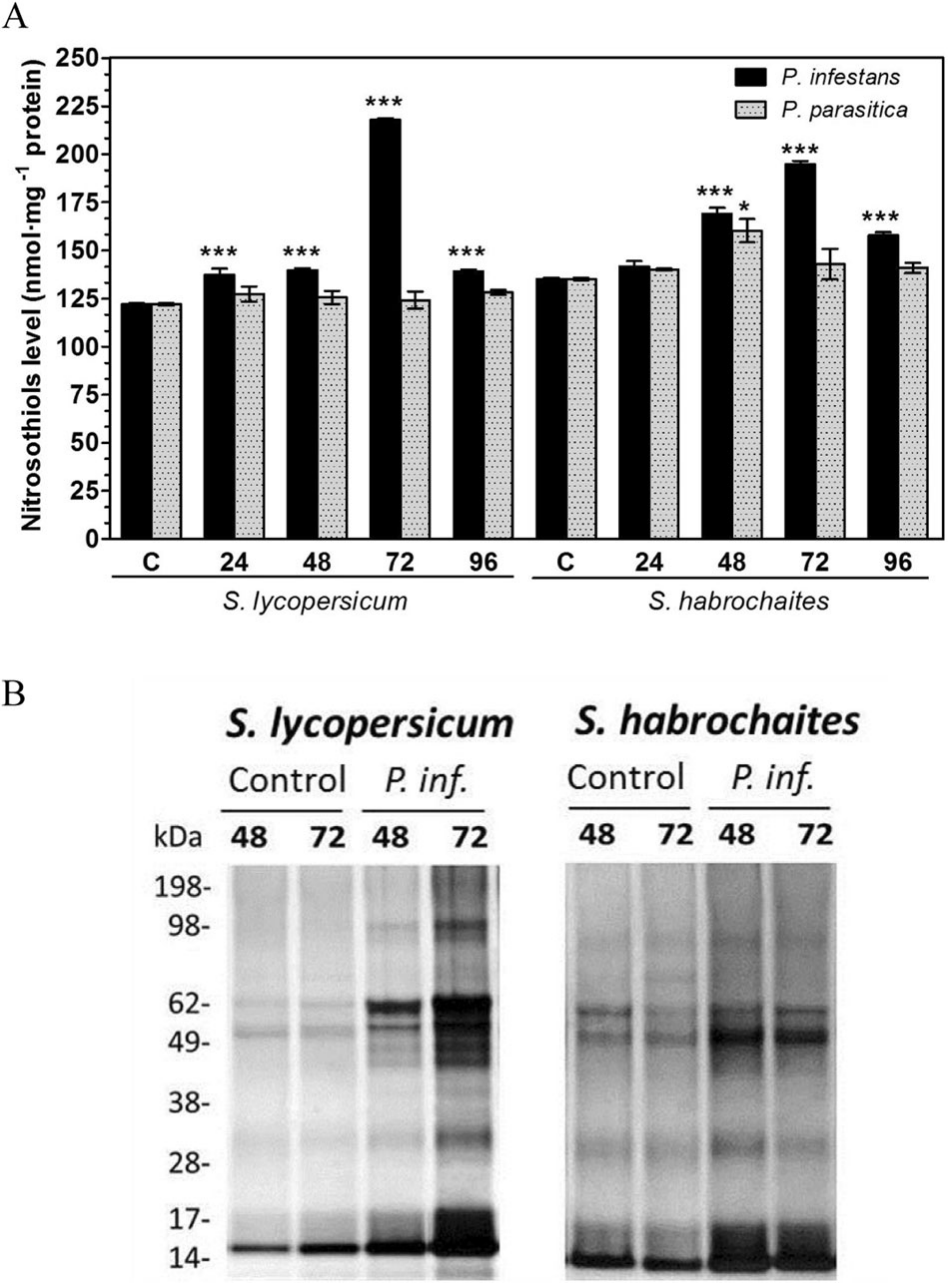
Analysis of protein S-nitrosothiols in leaf extracts during *P. infestans* and *P. parasitica* pathogenesis on *Solanum* spp. genotypes. **A** The content of protein S-nitrosothiols in purified leaf extracts was analysed using a modified Saville colourimetric method at *λ* = 540 nm. **B** Purification of S-nitrosated proteins in response to *P. infestans* infection. Totally, 10 mg proteins from tomato leaves inoculated with *P. infestans* were used for the biotin switch analysis and biotinylated proteins isolated using a neutravidin matrix. Eluates were separated by SDS-PAGE and proteins detected by silver staining (1 μg of protein per lane). The molecular masses of protein standards are shown on the *left*. Data represent means ± SD (*n* ≥ 3). Means significantly different from control are denoted by asterisks (ANOVA, *P < 0.05, ***P < 0.001)

Tomato leaves inoculated with *P. infestans* showed gradually increasing levels of protein S-nitrosothiols, which peaked at 72 hpi increased by approx. 80 and 45 % compared to control plants of *S. lycopersicum* and *S. habrochaites*, respectively, whereas *P. parasitica* pathogenesis did not cause a similar increase of protein S-nitrosothiols content, except for a slight increase in *S. habrochaites* at 48 hpi (Fig. 3A). We exploited the biotin-switch technique (BST) to analyse the patterns of S-nitrosated proteins in extracts of control and infected *Solanum* spp. plants (Supplementary Fig. S2). The measured levels of S-nitrosated proteins increased in leaf extracts of plants treated with both pathogens *P. infestans* and *P. parasitica*. Utilisation of various negative controls confirmed the specificity of the method (Supplementary Fig. 2C) and it was considered suitable for analysis of protein S-nitrosation patterns during biotic stress of both tomato genotypes. Biotin-labelled protein S-nitrosothiols were purified by affinity chromatography and analysed by sodium dodecyl sulfate-polyacrylamide gel electrophoresis (SDS-PAGE) and in-gel protein silver staining (Fig. 3B). Observed patterns of purified S-nitrosated proteins indicated significant modulation by *P. infestans* infection since a higher amount of S-nitrosated proteins was purified from leaves of susceptible genotype infected with *P. infestans* namely at 48 and 72 hpi (Fig. 3B). As expected, no differences in purified S-nitrosated proteins were observed in tomato leaf extracts from *P. parasitica*-treated plants in comparison to control plants (Supplementary Fig. S3).

**Fig. 4:**
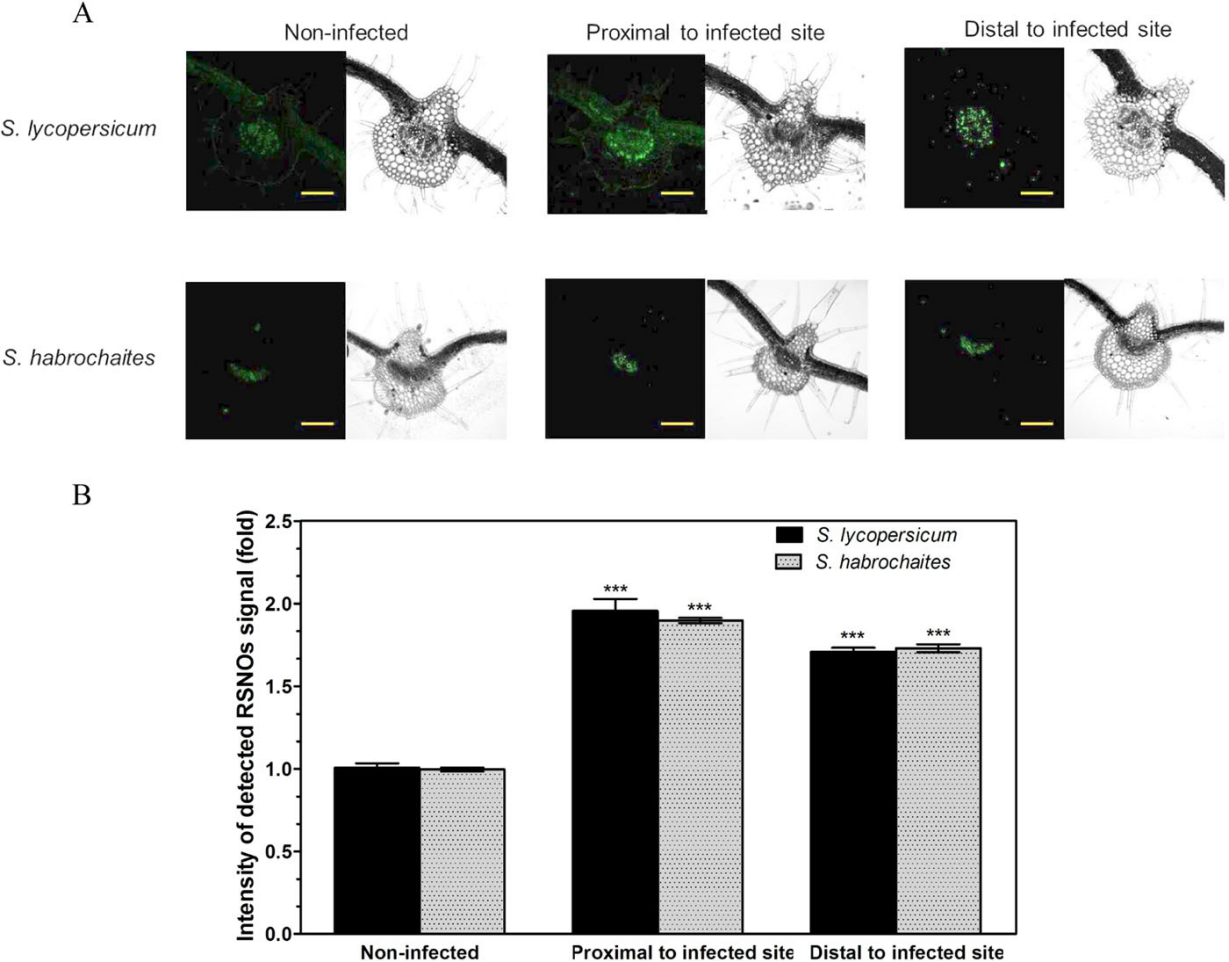
Detection of S-nitrosothiols by in *Solanum* spp. 72 hpi with *P. infestans*. **A** S-nitrosothiols (RSNO) were localised in cross-sections of *S. lycopersicum* and *S. habrochaites* infected leaves using 10 μM Alexa Fluor 488 Hg-link (scale bar = 200 μm). **B** Quantification of fluorescence signal intensity was performed by ImageJ 1.33 software using a non-infected control plant of each genotype as a reference value (=1). Data represent means ± SD (*n* ≥ 3). Means significantly different from the control are denoted by asterisks (ANOVA, ***P < 0.001)

Tissue distribution of S-nitrosothiols was studied in cross-sections of tomato leaves 72 hpi by CLSM using a specific fluorescent probe Alexa Fluor® Hg-Link phenylmercury (Fig. 4). Similarly to GSNOR, S-nitrosothiols in control non-infected leaves of both tomato genotypes localised to leaflet vascular bundles, parenchyma cells and epidermis (Fig. 4A). A significant increase in fluorescence signal corresponding to S-nitrosothiols was detected 72 hpi in both genotypes in the vascular bundles proximal and distal to the site of *P. infestans* infiltration. No significant differences in the intensity of S-nitrosothiol and GSNOR fluorescence signals were found in between leaf parts proximal and distal to the infection site, respectively (Fig. 4B). Data for *P. parasitica* are not presented as no significant changes were observed during studied stages of its pathogenesis.

### Proteomic analysis of S-nitrosated proteins in tomato leaves

**Fig. 5:**
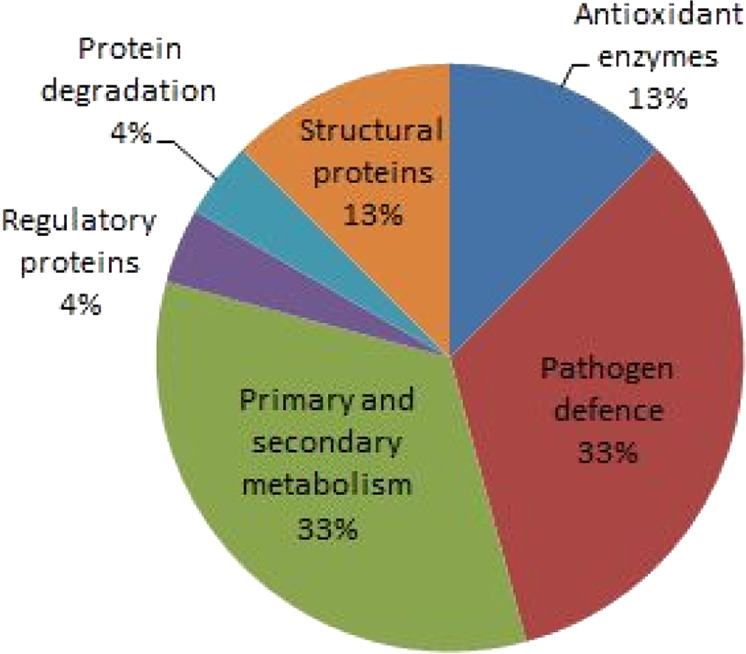
Classification of S-nitrosated proteins identified by the proteomic analysis into functional categories. Proteins found specifically S-nitrosated in both tomato genotypes infected with *P. infestans* 72 hpi were classified into corresponding functional categories as listed in Table 1

The affinity-purified protein fraction of biotin-labelled S-nitrosated proteins from the leaves 72 hpi with *P. infestans* and non-infected controls were subjected to 2D PAGE separation, trypsin in-gel digestion and liquid chromatography–mass spectrometry (LC–MS)/MS analysis. S-nitrosated proteins identified specifically in infected leaves were categorised according to their functions, with an indication of the samples in which corresponding proteins were detected and previous evidence for S-nitrosation (Table 1, Fig. 5). Detailed proteomic information (MW, pI, sequence coverage, etc.) for identified S-nitrosated proteins is described in Supplementary Table 1.

**Table 1:** S-nitrosated proteins identified in tomato leaves 72 hpi with *Phytophthora infestans*

Identified proteins	NCBI reference sequence	*S. lycopersicum* cv. Amateur	*S. habrochaites*	Cys-NO site predicted by GPS-SNO 1.0	Cys-NO site predicted by iSNO-PseAAC	References
*Antioxidant enzymes*						
Ascorbate peroxidase	CAB58361.1	+	+	Cys4	**Cys32** Cys138 Cys213	Clark et al.^75^; Lin et al.^76^; Tanou et al.^77^; Correa-Aragunde et al.^37,78^; de Pinto et al.^79^, Begara-Morales et al.^4,9^; Yang et al.^38^; Jain et al.^64^
Dehydroascorbate reductase 1	AAY47048.1	+	−	Cys6 **Cys20**	−	Fares et al.^80^; Kato et al.^81^; Puyaubert et al.^39^
Monodehydroascorbate reductase (NADH)-like protein	NP_001295285.1	+	−	Cys177	Cys67 Cys68 Cys142	Fares et al.^80^; Puyaubert et al.^39^; Begara-Morales et al.^4^; Jain et al.^64^
*Defence proteins and enzymes*						
Basic 30 kDa endochitinase precursor	NP_001234403.1	+	−	−	Cys93 Cys176 Cys199 Cys239	−
Heat shock 70 kDa protein 15-like	XP_004252333.1	+	+	Cys709	Cys40 Cys140 Cys209 Cys268 Cys368 Cys709 Cys780	Lindermayr et al.^3^; Abat et al.^82^; Maldonado-Alconada et al.^44^; Cheng et al.^83^
Heat shock protein 83	XP_004234218.1	+	−	Cys549 Cys574 Cys575	Cys350 Cys549 Cys575	Lindermayr et al.^3^; Abat et al.^82^; Maldonado-Alconada et al.^44^
Chaperone protein ClpB1 (Hsp101)	XP_004235966.1	+	−	Cys312	Cys312	Maldonado-Alconada et al.^44^; Jain et al.^64^
Putative late blight resistance protein homologue R1B-16	XP_004243044.2	−	+	Cys227 Cys382 Cys401 Cys462 Cys854	Cys223 Cys419 Cys719	−
S-adenosylmethionine synthase 1	NP_001234425.1	+	−	−	Cys45 Cys161	Lindermayr et al.^6^
S-adenosylmethionine synthase 2	NP_001296305.1	−	+	Cys20	Cys161	
Thioredoxin peroxidase 1 (Prx1)	NP_001234171.1	+	−	−	Cys76	−
*Enzymes of primary and secondary metabolism*						
ATP synthase subunit alpha, mitochondrial	XP_004253362.1	+	−	−	Cys201	Lindermayr et al.^3^; Tanou et al.^84^; Puyaubert et al.^39^
ATP synthase CF1 alpha subunit, chloroplast	YP_008563073.1	+	+	−	Cys194	Eaton et al.^85^; Lindermayr et al.^3^
Caffeoyl-CoA O-methyltransferase	NP_001234801.1	+	+	−	Cys238	−
Enolase	NP_001234080.1	+	−	Cys408	Cys107 Cys124 Cys191 Cys346	Lindermayr et al.^3^; Romero-Puertas et al.^56^; Fares et al.^80^; Correa-Aragunde et al.^37^; Jain et al.^64^
Enolase-like	NP_001332774.1	−	+	Cys408	Cys107 Cys124 Cys191 Cys346	
Glyceraldehyde-3-phosphate dehydrogenase, cytosolic	NP_001266254.2	+	−	**Cys156** **Cys160** Cys333	−	Holtgrefe et al.^5^; Veskovi et al.^86^; Wawer et al.^87^; Henry et al.^88^; Testard et al.^89^; Romero-Puertas et al.^56^; Tanou et al.^90^; Bedhomme et al.^91^; Zaffagnini et al.^92^; Jain et al.^64^
Glyceraldehyde-3-phosphate dehydrogenase A, chloroplastic	XP_004236849.1	−	+	Cys216	Cys82	−
Triosephosphate isomerase, cytosolic	XP_004236746.1	+	−	Cys13 Cys127	Cys13 Cys67 Cys127	Jain et al.^64^
*Regulatory proteins*						
14-3-3-like protein	XP_004234565.1	+	−	Cys99	Cys99 Cys194	Lindermayr et al.^3^; Doulias et al.^93^; Fares et al.^80^; Jain et al.^64^
*Protein degradation*						
26S protease regulatory subunit 6 A homologue	P54776.1	+	+	−	Cys105 Cys224 Cys265	−
*Structural proteins*						
Actin-41	NP_001317048.1	+	−	Cys12 Cys287	Cys12 Cys259	Lindermayr et al.^3^
Actin-7	NP_001295376.1	−	+	Cys12 Cys287	Cys12 Cys259	
Actin	NP_001309932.1	−	+	Cys12 Cys287	Cys12 Cys259	

Leaves of *S. lycopersicum* cv. amateur and *S. habrochaites* were inoculated with *P. infestans* and collected 72 hpi together with corresponding non-infected controls. Leaf extracts were analysed by the biotin switch procedure and affinity-purified biotinylated proteins were subsequently analysed by 2D-PAGE and LC–MS/MS analysis as described in “Methods”. The proteins identified as S-nitrosated in vivo in respective tomato genotypes due to infection are marked as +. NO-sensitive cysteine residues predicted using the GPS-SNO 1.0 software (medium threshold) and iSNO-PseAAC and previously confirmed as S-nitrosated in vitro in cited references are marked in bold.

Several proteins were found in both genotypes as common S-nitrosation targets, including cytoskeletal protein actin, metabolic proteins, glycolytic enzymes GAPDH and enolase, caffeoyl-CoA *O*-methyltransferase involved in phenylpropanoid biosynthesis, and methionine-adenosyl transferase 1 and 2, key enzymes of ethylene biosynthesis. Ascorbate peroxidase (APX, EC 1.11.1.11), a key antioxidant enzyme of the ascorbate–glutathione cycle, was also found S-nitrosated after *P. infestans* infection in both genotypes. Proteins with regulatory functions (14-3-3-like proteins) as well as proteins involved in plant defence mechanisms (basic chitinase, Hsp70, Hsp83, Hsp101, thioredoxin peroxidase 1, monodehydroascorbate and dehydroascorbate reductase) were among the most prominent targets of S-nitrosation in the *S. lycopersicum* genotype. Interestingly, the putative late blight resistance protein homologue R1B-16 was identified only in the resistant genotype *S. habrochaites* (Table 1).

GPS-SNO 1.0 and iSNO-PseAAC software tools were employed to predict the occurrence of nitrosation-sensitive cysteine residues in S-nitrosated proteins identified by the proteomic analysis. However, we did not observe a satisfactory degree of agreement in prediction results, except for structural protein actin, where Cys12 were predicted by both software tools in all three actin isoforms (Table 1).

### APX protein levels and activity are increased by *P. infestans* infection

**Fig. 6:**
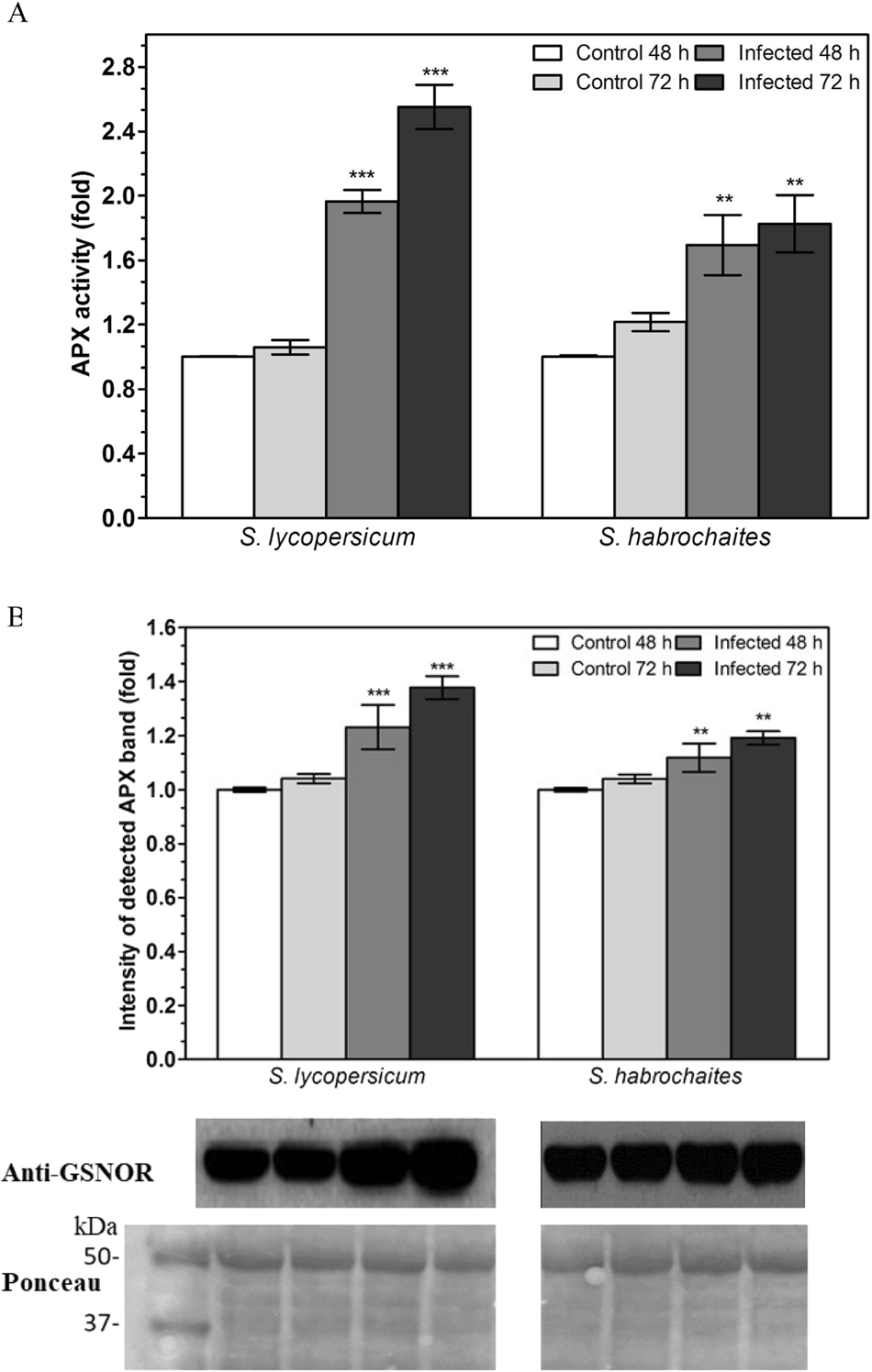
Modulation of APX activity and protein level during the pathogenesis of *P. infestans*. **A** APX activity in leaf extracts was evaluated spectrophotometrically at 25 °C by monitoring the oxidation of ascorbate in the presence of H_2_O_2_ at *λ* = 290 nm. **B** APX protein levels were quantified in leaf samples (100 μg of protein per lane) inoculated with *P. infestans* 48 and 72 hpi by Western blot analysis using anti-APX polyclonal rabbit antibody (dilution 1:2000) and goat anti-rabbit IgG peroxidase conjugate (dilution 1:10,000). The intensity of bands was quantified by ImageJ 1.33 software using a non-infected control plant of each genotype as a reference value (=1). Data represent means ± SD (*n* ≥ 3). Significantly different means from the control are denoted by asterisks (*t* test, **P < 0.01, ***P < 0.001). Reversible Ponceau staining was used as a loading control. The molecular masses of protein standards are shown on the *left*

Based on the data obtained by mass spectrometry (Table 1) where APX was identified as a significant target of S-nitrosation after *P. infestans* infection in both genotypes, we analyse changes in APX activity and protein level during the course of *P. infestans* pathogenesis (Fig. 6). APX activity was evaluated by spectrophotometric measurement of H_2_O_2_-dependent ascorbate oxidation while the level of APX protein was determined by immunoblot analysis using an APX-specific antibody. APX enzyme activity and protein level were found significantly increased at 48 and 72 hpi in both genotypes, with a more pronounced increase in susceptible *S. lycopersicum*. Interestingly, the relative increase of APX activity in infected plants compared to controls (Fig. 6A) was higher than the corresponding increase in APX protein level (Fig. 6B), suggesting a significant modulation of APX activity by post-translational modifications.

## Discussion

In comparison to cultivated tomato genotypes, where the genetic background is particularly lacking for resistance to *P. infestans*, wild *S. habrochaites* genotype contains genes for resistance to most important tomato diseases including late blight and can be used as a potential source of quantitative resistance^29^. Inbred lines resulting from an interspecific cross between *S. lycopersicum* and *S. habrochaites* f. glabratum also exhibited resistance to numerous *P. infestans* isolates^30^. In our preliminary testing, *S. habrochaites* also showed a high level of basal resistance to *P. parasitica*, unlike susceptible *S. lycopersicum* cv. Amateur (J. Lochman, personal communication).

Previously, we have uncovered specific roles of NO in tomato powdery mildew pathogenesis in studies on cultivated and wild tomato genotypes differing in their resistance^22,24,27^. Protein S-nitrosation (referred also to as protein S-nitrosylation) has emerged as a key NO-dependent signalling pathway in plant responses to pathogen infection^31,32^. In this study, significant modulations of protein S-nitrosothiol levels and GSNOR activity were observed up to 96 hpi in *Solanum*–*P. infestans* interactions, whereas only minor changes were recorded following inoculation with *P. parasitica* (Fig. 1). This is in accordance with observations of increased S-nitrosothiols due to decreased GSNOR activity in Arabidopsis non-host resistance to *P. syringae* pv. *tomato*^18,19^ and sunflower resistance to *Plasmopara halstedii*^8^.

Recent analysis of the tomato nitrosoproteome confirmed increased endogenous NO level and S-nitrosation in tomato GSNOR-knockdown plants, resulting in increased germination, decreased root and hypocotyl growth, photosynthesis and plant growth together with low fructification and fruit yield^16^. It has been concluded that S-nitrosation of crucial proteins in the plant energy metabolism mediated by GSNOR has a decisive role in the regulation of tomato growth and development. Another recent study on tomato revealed that reduced abundance of GSNOR transcript compromised SA biosynthesis and signalling, resulting in decreased basal resistance, whereas increased expression of tomato GSNOR enhanced pathogen protection^17^. However, our recent study showed that in *Solanum* spp.—*Pseudoidium neolycopersici* pathosystem reduced GSNOR activity and GSNO accumulation was related to a higher degree of tomato resistance to powdery mildew^24^. The observed discrepancies in modulations of plant GSNOR in response to pathogens might be related to diverse lifestyle and infection strategies among bacterial, fungal and oomycete phytopathogens. Interestingly, higher NO production and decreased GSNOR activity were observed in lower leaves of susceptible potato genotype (*Solanum tuberosum* cv. Bintje) following SAR induction by BABA, INA and laminarin, whereas a combined treatment with SAR inductors and *P. infestans* infection led to an opposite effect, i.e. increased GSNOR activity and lower S-nitrosothiol levels^33^.

Our results show S-nitrosothiols localised mainly in the veins which suggest their signalling role not only in proximal but also in distal parts of the infected leaflets, in agreement with suggested signalling role of NO in tomato defence to powdery mildew^22^. In olive leaves, S-nitrosothiols were localised into the phloem whereas GSNO in the vascular bundles and mesophyll^34^. Following infection with *P. halstedii*, a significant increase in S-nitrosothiols was observed in epidermal, parenchymal and cortical cells of the susceptible sunflower genotype, but only in epidermal cells and the vascular bundle of the resistant genotypes, whereas GSNOR signal was amplified in cortical and epidermal cells of the hypocotyl^8^.

We utilised the biotin-switch technique (BST) as a specific proteomic approach using reversible labelling of S-nitrosated cysteines with a biotin tag^35^. By comparing the data from our analysis of proteins S-nitrosated in plants infected with *P. infestans* 72 hpi with previous reports, a number of common target proteins subjected to S-nitrosation have been uncovered, including antioxidant enzymes, defence proteins, enzymes of primary and secondary metabolism, and regulatory and structural proteins (Table 1). The ascorbate–glutathione cycle represents a crucial antioxidant mechanism that protects cells from oxidative damage^36^. Enzymes of the ascorbate–glutathione cycle, namely APX, represent important S-nitrosation targets in plants^4,9,37,38^. In our study, S-nitrosated APX was identified in both tomato genotypes after infection with *P. infestans* (Table 1). Relatively higher increase of APX activity compared to an increase of APX protein level observed at 48 and 72 hpi suggest post-translational regulation of APX activity such as S-nitrosation (Fig. 5). Higher increase of both APX activity and protein in the susceptible *S. lycopersicum* genotype, namely at 72 hpi, might be related to a higher level of oxidative stress, associated with the transition from a biotrophic to the necrotrophic phase of *P. infestans* pathogenesis. Previous in vitro and in vivo studies showed S-nitrosation of Cys32 in pea cytosolic APX resulted in increased enzyme activity^9^. Similarly, S-nitrosation of Cys32 and its positive effect on APX activity was confirmed in Arabidopsis^38^. S-nitrosation of dehydroascorbate and monodehydroascorbate reductases were found in *S. lycopersicum* (Table 1), similarly to Arabidopsis^39^ and pea peroxisomes^4^. Previously, we observed that the activity of APX was significantly increased in the roots of both *Solanum* spp. genotypes cultivated in agar medium supplemented with GSNO or GSNOR inhibitor N6022, which was associated with increased S-nitrosation of APX^40^. In this study, the increase of GSNOR activity induced by salinity and cadmium stress in *S. habrochaites* and subsequent lower levels of S-nitrosothiols resulted in decreased S-nitrosation status and decreased APX activity, in contrast to stress-induced down-regulated GSNOR activity in *S. lycopersicum* cv. Amateur which increased levels of S-nitrosothiols and APX S-nitrosation and activity. Collectively, responses of even closely related plant genotypes to abiotic stresses and pathogen infection might significantly differ in diverse modes of modulations of key components of ROS and RNS metabolism.

Here, we have identified pathogen-triggered S-nitrosation of specific defence proteins, including basic endochitinase precursor and late blight resistance protein homologue R1B-16, which have not been previously described as S-nitrosation targets. Within plant defences to pathogens, pathogen-related (PR) proteins represent a heterogeneous group of proteins involved in plant responses to viral, bacterial and fungal pathogens^41^. A 30 kDa basic endochitinase (EC 3.2.1.14), identified as an S-nitrosation target in susceptible *S. lycopersicum*, belongs to PR-3 proteins and catalyses a hydrolytic cleavage of β(1 → 4) glycosidic linkages of chitin and chitodextrins. Tobacco class I chitinase possesses an N-terminal cysteine-rich domain; however, this domain was shown indispensable for the binding of chitin but not for its catalytic or antifungal activity^42^.

In both *Solanum* spp. genotypes, S-nitrosation of heat shock proteins 70 (HSP70) was observed, whereas S-nitrosated HSP83 and HSP101 were detected only in the susceptible genotype (Table 1). HSPs are evolutionarily highly conserved molecular chaperones expressed in low levels constitutively but highly induced under stress conditions^43^. S-nitrosation of the Hsp70 and Hsp100 family were previously reported under physiological and stress conditions^3,44^. In animal studies, S-nitrosation of Cys597 in HSP90 promotes inhibition of its ATPase activity and reduced the ability to activate endothelial NOS^45^, whereas S-nitrosation of Cys66 in mitochondrial HSP70 modulates its chaperon function^46^. Although highly conserved structures and functions of HSP families suggest similar S-nitrosative modifications of HSP function to operate in plants, this possibility nevertheless requires experimental verification.

For both tomato genotypes, 26S proteasome regulatory subunit 6A was identified as S-nitrosation target (Table 1). The 26S proteasome participates in the ATP-dependent degradation of ubiquitinated proteins and the regulatory subunit provides ATP dependency and substrate specificity to the proteasome complex^47^. In animals, ubiquitin-dependent 26S proteasome degradation is inactivated by S-nitrosation, which contributes to protein aggregation and neurodegenerative disorders^48^. In plants, the ubiquitin/26S proteasome system is involved in defences to a wide array of pathogens, where it is required in jasmonate, SA and ethylene signalling pathways^49^. SGT1, a representative of E3 ubiquitin ligases responsible for the final protein tagging, has a universal role in diverse plant defence responses mediated by R genes. SGT1 is involved in potato resistance to *P. infestans* triggered by a resistance gene, RB, cloned from a wild potato *Solanum bulbocastanum*^50^, however functional implications of S-nitrosation of the 26S proteasome regulatory subunit in plant responses to pathogen infection remains to be elucidated.

Interestingly, putative late blight resistance protein homologue R1B-16 was found as the S-nitrosation target in the resistant genotype *S. habrochaites* (Table 1). This protein confers resistance to *P. infestans* races carrying the avirulence gene *Avr1* (ref. ^51^). Interactions of resistance proteins with avirulence factors trigger plant defence mechanisms including the hypersensitive response, which restricts the growth of biotrophic pathogens^52^. It has been shown that immune activation mediated by the late blight resistance protein R1 requires its nuclear localisation^53^. S-nitrosation might be involved in mechanism controlling R1 protein intracellular localisation, similarly to other defence-related proteins and transcription factors^44^.

S-nitrosation of methionine adenosyltransferase (MAT, EC 2.5.1.6) is known to exert significant metabolic effects through changes in S-adenosylmethionine-dependent methylation reactions, biosynthesis of plant growth regulators polyamines and ethylene and gene expression control. Three isoforms MAT1, MAT2, MAT3 undergoing S-nitrosation were previously found in plants^6^. In our pathosystem, MAT1 isoform in *S. lycopersicum* and MAT2 isoform in *S. habrochaites* were identified S-nitrosated after infection with *P. infestans* (Table 1). Incubation of MAT1 with GSNO in vitro results in a 30% loss of enzyme activity due to Cys114 S-nitrosation, whereas S-nitrosation of MAT2 and MAT3 isoforms does not affect their activity^6^. Reversible S-nitrosation of MAT1 leads to a reduction in S-adenosylmethionine and consequently ethylene levels, indicating a role of MAT1 as a molecular switch in cross-talk of ethylene and NO signalling in the *Solanum*–*Phytophthora* pathosystem.

Thioredoxins (TRX) are a group of small proteins containing two Cys residues in the active site, showing oxidoreductase activity which regulates structure and function of their protein targets. TRX in conjunction with peroxiredoxins (Prx, EC 1.11.1.15) acting as TRX-dependent peroxidases scavenges H_2_O_2_ and peroxynitrite^54^. Similarly to animal TRXs, some plant TRX isoforms are involved in direct denitrosation of specific proteins. Within Arabidopsis immunity, TRXh5 was shown to reverses protein S-nitrosation acting as a selective protein-SNO reductase^55^. In our study, S-nitrosated Prx1 was identified after infection with *P. infestans* in the susceptible genotype *S. lycopersicum* (Table 1). In Arabidopsis leaves, cytosolic PrxII B and chloroplast PrxII E isoforms were S-nitrosated within the plant immune responses^3,56^. During the Arabidopsis hypersensitive reaction to *P. syringae* pv. *tomato*, S-nitrosation of PrxII E was recorded during defence response, which inhibited the peroxidase and peroxynitrite reductase activity of PrxII E. Prxs are both S-nitrosation and carbonylation targets, and S-nitrosation of the active Cys residue can lead to conformational changes that prevent carbonylation and subsequent irreversible depletion of protein functionality, as described during salinity stress in pea mitochondria^57^.

Activities of both mitochondrial and chloroplast ATP synthases within cellular bioenergetics machinery are regulated by redox modifications of key Cys residues^58^. We identified the subunit α of chloroplast CF1 ATP synthase as another S-nitrosation target in both tomato genotypes after *P. infestans* infection. In addition, in susceptible *S. lycopersicum*, S-nitrosation of the α-subunit of mitochondrial ATP synthase was detected (Table 1). In animals, nitrosative or oxidative modifications of Cys294 located on the surface of the α subunit of ATP synthase can serve as a redox-modulator of cellular ATP levels^59^. S-nitrosation of ATP synthase in intact rat brain mitochondria by GSNO inhibits its activity^58^. Endogenous S-nitrosation of the α subunit of chloroplast ATP synthase was already reported in Arabidopsis^3,10^. We hypothesise that increased S-nitrosation of ATP synthase subunits may result from pathogen-triggered oxidative stress and subsequent depletion of mitochondrial or chloroplast glutathione, as described in oxidative-stress associated dysfunctions in animals^58^.

In both tomato genotypes, we identified S-nitrosated caffeoyl-CoA-O-methyltransferase (EC 2.1.1.104), which catalyses the transfer of a methyl group from S-adenosylmethionine to caffeoyl-CoA to produce feruloyl-CoA, a key reaction in the lignification, a secondary cell wall reinforcement to increase plant resistance^60^. NO modulates the activity and expression of several enzymes of lignin biosynthesis: phenylalanine ammonium lyase (EC 4.3.1.24) and peroxidases (EC 1.11.1.7) are induced in soybean seedlings by a NO donor^61^. Maize *trans*-cinnamate 4-monooxygenase (EC 1.14.13.11), an important enzyme of phenylpropanoid biosynthesis, is inhibited by NO binding to the cytochrome P450 heme group^62^. Moreover, a transcriptomic study identified endogenous NO as a transcriptional regulator of genes of lignin biosynthesis in sunflower roots^63^. NO also indirectly regulate the lignin biosynthesis through transcriptional control of enzymes involved in the production of H_2_O_2_ which serves as a substrate to peroxidases-catalysed polymerisation of monolignols. Furthermore, NO modulates antioxidant enzymes involved in the removal of H_2_O_2_ through the ascorbate–glutathione cycle^4,9,38^.

In both genotypes, enolase (EC 4.2.1.11) was S-nitrosated after infection with *P. infestans* (Table 1). Enolase, catalysing the conversion of 2-phosphoglycerate to phosphoenolpyruvate, was previously identified as an S-nitrosation target in Arabidopsis^3,37^ and sunflower^64^. In the susceptible genotype, we also detected S-nitrosated triosephosphate isomerase (EC 5.3.1.1), which catalyses a reversible conversion of glyceraldehyde-3-phosphate to dihydroxyacetone phosphate. This enzyme was found endogenously S-nitrosated under normal growth conditions in Arabidopsis WT Col-0 and *gsnor1-3* mutant lines^10^. There is currently no information available on the functional relevance of S-nitrosative modifications of plant glycolytic enzymes. Recently, the first study on S-nitrosation of human triosephosphate isomerase found a 30% decrease of the *V*_max_ value in the enzyme S-nitrosated at Cys271, without affecting the *K*_M_ for its substrate dihydroxyacetone phosphate^65^.

In our study, *P. infestans* pathogenesis induced S-nitrosation of another glycolytic enzyme, cytosolic glyceraldehyde-3-phosphate dehydrogenase (GAPDH, EC 1.2.1.12) in *S. lycopersicum* and its chloroplast isoform A in *S. habrochaites* (Table 1). Cytoplasmic GAPDH isoforms in Arabidopsis and tobacco, as well as pea cytosolic GAPDH and chloroplastic A2B2-GAPDH, were previously reported to undergo stress-mediated re-localisation to the nucleus triggered by S-nitrosation of Cys155 and Cys159 (ref. ^5^). Recently, it was confirmed both in vitro and in vivo that NO affects directly the activity of Arabidopsis GAPDH plastid isoforms by S-nitrosation^66^. In overall, our findings suggest S-nitrosation-mediated inhibition can contribute to glycolysis impairment during oxidative/nitrosative conditions induced within plant defence responses to pathogenic challenge.

In *S. lycopersicum*, the 14-3-3-like protein was identified as S-nitrosated 72 hpi after *P. infestans* infection. The epsilon isoform of the 14-3-3 family was previously found S-nitrosated in response to a NO donor in Arabidopsis^3^. Recently, it was demonstrated that the epsilon isoform in Arabidopsis interacts specifically with a number of ribosomal proteins suggesting an important function in protein synthesis^67^. Cysteine S-nitrosation thus can constitute a common regulatory mechanism for the protein synthesis mediated by 14-3-3 proteins in plants under biotic stress conditions.

Rearrangements of actin filaments and microtubules belong to the earliest stress responses involved in translocation of organelles and structural defence mechanisms at the site of pathogen penetration, e.g. deposition of biopolymers reinforcing plant cell walls^68^. In both genotypes, actin was identified as an S-nitrosation target after *P. infestans* infection. The role of NO as a modulator of the actin cytoskeleton has been previously suggested in maize roots^69^ and conifer pollen^70^. S-nitrosation of actin, α- and β- tubulin was firstly reported in *A. thaliana*^3^. In Arabidopsis leaves exposed to 2,4-dichlorophenoxy acetic acid both oxidation and S-nitrosation of actin were previously detected resulting in a reduction in the F-actin/G-actin ratio^71^.

Collectively, the results obtained in our studies of *Solanum*–*Phytophthora* spp. interactions demonstrate that elevated levels of NO and S-nitrosothiols during infection by hemibiotrophic pathogen have an important regulatory role in defence mechanisms mediated by S-nitrosation of key defence and metabolic enzymes. Moreover, localisation of S-nitrosothiols as relative stable NO metabolites to vascular bundles suggests their involvement in the systemic signal transduction within infected tomato plants. Using proteomic analysis, S-nitrosation targets were identified in both tomato genotypes 72 hpi by *P. infestans*, including proteins of primary and secondary metabolism, structural proteins, proteins involved in signalling and defence mechanisms. Among them, ascorbate peroxidase activity was found activated post-translationally by S-nitrosation in infected leaves of both tomato genotypes.

## Materials and methods

### Plant material and growth conditions

*S. lycopersicum* cv. Amateur and *S. habrochaites* f. *glabratum* (LA 2128), i.e. genotypes susceptible and resistant to powdery mildew, respectively, were used^22^. Seeds were germinated in perlite, seedlings transferred to a mixture of garden soil/peat (2:1, v/v) and grown in a glasshouse at 22/18 °C, 12/12 h (day/night) regime. Plants were inoculated at the age of 50 days.

### Pathogen material and maintenance

*P. infestans* (Mont.) de Bary, strain VŽ 14/14 (obtained from J. Mazáková, Czech University of Life Sciences, Prague) and *P. parasitica* Dastur, strain 149 (obtained from J. Lochman, Masaryk University, Brno) were maintained on V8 juice agar (Čaderský-Envitek, Brno, Czech Republic). The cultures were grown at 25 °C under a 12 h photoperiod and sub-cultivated every 2–3 weeks.

### Pathogen inoculation and incubation

Inoculations by both *P. infestans* and *P. parasitica* were performed by infiltrating the parenchyma of leaf blades with 50 μl suspensions containing 100 zoospores or with 50 μl of deionized water (control). In each plant, three leaflets per each of 4th to 7th odd-pinnate leaves were infiltrated by the zoospore suspension as indicated in Supplementary Fig. 1a. All inoculation procedures were started at 8:30 a.m. to follow the same circadian rhythm during plant treatments and sampling. Leaf samples were harvested 0, 24, 48, 72 and 96 h post inoculation (hpi) and either fixed for histochemical or immunohistochemical staining or frozen in liquid nitrogen and stored at −80 °C.

### Preparation of plant extracts

One gram of excised leaf samples was homogenised in liquid nitrogen using a mortar, pestle and the extraction buffer (50 mM Tris-HCl, 2 mM dithiothreitol, 0.2% Triton X-100, 1 mM phenylmethylsulfonyl fluoride, pH 7.5). Leaf homogenates were centrifuged at 16,000x*g* at 4 °C for 30 min and supernatants purified using NAP-10 columns (GE Healthcare, USA).

### Measurement of protein S-nitrosothiols

Protein S-nitrosothiol content was determined in purified leaf extracts by modified Saville assay^35^. Purified extracts (5 μl) were incubated for 5 min with 100 μl of 3.5% sulphanilamide in 0.5 M HCl or 100 μl of 3.5% sulphanilamide in 0.5 M HCl containing 1% HgCl_2_ in 96-well microplates. After addition of 100 μl of 0.1% N-(1-naphthyl)-ethylenediamine dihydrochloride in deionized water and 5 min incubation, the formation of the reaction product was followed by measurements of absorbance changes at 540 nm (Synergy HT, BioTek Instruments, USA). S-nitrosothiols were determined as the difference of absorbance between values obtained with and without added HgCl_2_, using a standard curve prepared with synthetic GSNO. The values were expressed per milligram of total protein measured by the Bradford method^72^.

### Enzyme activity of GSNOR and APX

GSNOR activity was determined spectrophotometrically in freshly prepared and purified extracts of plant leaves by measurements of NADH absorbance at 340 nm^28^. APX activity was determined by monitoring the velocity of ascorbate oxidation in the presence of H_2_O_2_ determined by changes of the absorbance at 290 nm^9^.

### Preparation of leaflet cross-sections

Central parts of tomato leaflets both distal and proximal to infected sites (see Supplementary Fig. 1b) were sliced into 5 × 5 mm pieces and fixed for 3 h in 4% paraformaldehyde in 0.1 M phosphate buffer, pH 7.4, at laboratory temperature. Serial 80 µm sections were obtained by a vibratome (Leica VT1000S, Leica Biosystems, Germany) upon mounting in 4% agarose.

### Histochemical detection of S-nitrosothiols

All procedures were done at laboratory temperature in the dark to minimise decomposition of light-sensitive S-nitrosothiols. Leaflet cross-sections were incubated with 10 mM N-ethylmaleimide (NEM) and 100 μM diethylenetriaminepentaacetic acid (DTPA) in ethanol for 1 h at 25 °C, washed three times 15 min in 10 mM Tris/HCl (pH 7.4), incubated with β-mercaptoethanol for 10 min, and then washed three times in 10 mM Tris/HCl, (pH 7.4). Samples were incubated with 10 µM Alexa Fluor 488 Hg-link phenylmercury (AF-Hg, Thermo Fisher Scientific, USA) for 1 h at 25 °C, washed three times in 10 mM Tris/HCl pH 7.4 and mounted on glass slides in glycerol and 10 mM Tris-HCl, pH 7.4 (1:1, v/v). Three negative controls were used: sections incubated (i) with NEM and β-mercaptoethanol without AF-Hg; (ii) with β-mercaptoethanol and AF-Hg without NEM; and (iii) with β-mercaptoethanol only^9^.

### Immunolocalization of GSNOR

Leaflet cross-sections were incubated for 10 min in TBSA-BSAT buffer (5 mM Tris-HCl buffer, 0.1% bovine serum albumin, 0.9% NaCl, 0.1% Triton X-100, 0.05% sodium azide, pH 7.6), and then incubated with rabbit polyclonal antibody against tomato GSNOR in 1:500 dilution in TBSA-BSAT at 4° overnight^28^. Negative controls were done without the primary antibody. Sections were washed several times with TBSA-BSAT and incubated for 1 h with goat anti-rabbit IgG (H + L) DyLight® 488 conjugate (Thermo Fisher Scientific, USA) at laboratory temperature. After several washes in TBSA-BSAT, sections were mounted on glass slides in 50% glycerol in 10 mM Tris-HCl, pH 7.4.

### Confocal laser scanning microscopy

Samples were examined by a microscope IX81 equipped with a confocal laser scanning unit FV1000 (Olympus Czech Group, Prague, Czech Republic). Transmission light images, in single images combined with Nomarski DIC filters, were acquired using a 405 nm excitation with a near-ultraviolet diode laser. The fluorescence channel was obtained simultaneously by excitation with a 488 nm line of an argon laser and signal detection using a 505–525 nm emission filter.

### Purification and detection of S-nitrosated proteins

S-nitrosated proteins were purified and detected using the biotin switch technique as described previously^3^. Frozen leaf samples (2 g) were extracted in ratio 1:2 (w/v) with 100 mM HEPES-NaOH pH 7.4 with 10 mM EDTA, 0.1 mM neocuproine, 1% Triton X-100 and protease inhibitor cocktail (Sigma-Aldrich, USA). Extracts were centrifuged at 16,000×*g* for 30 min at 4 °C, protein concentration determined by the Bradford method^72^ and adjusted to 1 mg/ml. Samples were incubated with 2.5% SDS and 20 mM methyl methanethiosulfonate for 30 min at 50 °C with frequent mixing to block free cysteine thiols, and residual reagents were eliminated by acetone precipitation. Precipitates were re-suspended in 1% SDS and biotinylated with 1 mM ascorbate and biotin-HPDP (Thermo Fisher Scientific, USA) at for 1 h in laboratory temperature in the dark. Following acetone precipitation, isolated proteins were subjected to Western blot analysis or affinity purification^3^.

### Proteomic analysis of S-nitrosated proteins

S-nitrosated proteins in sample fractions obtained by affinity purification were separated by 2D-PAGE, in-gel digested by trypsin and identified by LC–MS analysis. For isoelectric focusing, 80 μg of total protein in the sample buffer were loaded in triplicate to Immobiline DryStrip pH 3–11 NL, 7 cm (GE Healthcare, USA). Samples were rehydrated using passive sample application during 18 h. Isoelectric focusing was done on a PROTEAN IEF Cell (Bio-Rad, USA) and proteins stained in gel with SYPRO Ruby (Bio-Rad, USA). The gels were fixed for 60 min, washed in water, and imaged by a Pharos FX Plus Molecular Imager (Bio-Rad, USA). The gels were analysed by PDQuest software (Bio-Rad, USA). Spots detected specifically in samples of infected leaves in comparison to non-infected controls were selected for analysis and excised with an EXQuest Spot Cutter (Bio-Rad, USA). Gel pieces were destained and incubated with trypsin (sequencing grade, Promega, USA) for 2 h at 37 °C. Trypsin digests were analysed by LC–MS/MS analysis performed on UltiMate 3000 RSLCnano coupled to Orbitrap Elite (Thermo Fisher Scientific). Chromatographic separation was achieved on a reverse-phase column with a 40–70 min water/acetonitrile gradient. MS data were recorded in Orbitrap at resolution 60,000 at 400 m/z and MS/MS data after HCD fragmentation collected at 15,000 resolution at 400 m/z. MS/MS data were searched against the UniProtKB database for tomato using the Mascot search engine. Complete proteome database contained 34824 protein sequences in total; cRAP database of contaminants was used in parallel. Trypsin specific cleavage with two allowed miscleavages was set for all database searches. Carbamidomethylation (C) was set as a fixed whereas deamidation (N, Q) and oxidation (M) as variable peptide modifications.

### Quantification of GSNOR and APX protein by Western blotting

Proteins in plant extracts were analysed by SDS-PAGE electrophoresis in 12% gel using a Mini-Protean cell (Bio-Rad, USA) and transferred to 0.45-μm nitrocellulose membrane. Blots were incubated for 2 h in a blocking buffer and then overnight with polyclonal rabbit antibody raised to tomato GSNOR in 1:1000 dilution^28^, or anti-APX polyclonal rabbit antibody in 1:2000 dilution (Agrisera, Sweden). The membranes were washed six times for 10 min in 0.1% Tween-20 in TBS and then incubated for 2 h with goat anti-rabbit IgG conjugated with horseradish peroxidase (Sigma-Aldrich, USA) in 1:10,000 dilution. The membranes were washed for 1 h in 0.1% Tween-20 in TBS and then incubated for 5 min with a Western blotting luminol reagent (Santa Cruz Biotechnology, USA). The chemiluminescence was detected with a photographic film (GE Healthcare, USA). Chemiluminescence signal intensities were assessed using ImageJ 1.33 software (National Institute of Health, USA).

### Software prediction of S-nitrosated cysteine residues

Freely available web tools GPS-SNO1.0 (ref. ^73^) and iSNO-PseAAC^74^ were used. Protein amino acid sequences were submitted in the FASTA format and the medium threshold conditions were selected for the batch prediction tool of GPS-SNO1.0 software.

### Statistical analysis

Significant differences in mean values of measured parameters among studied genotypes were assessed by one-way ANOVA and Bonferroni Multiple-Comparison Test using GraphPad Prism5. Bars in Figs. 1–4 and Fig. 6 represent means ± SD of data from three independent biological experiments performed in triplicate for each experimental conditions and genotype. Student’s *t* test was utilised for pairwise comparisons.

## Supplementary information

Supplementary Figures

Supplementary Table S1

## References

[1] Kolbert Z (2019). A forty year journey: the generation and roles of NO in plants. Nitric Oxide.

[2] Umbreen S (2018). Specificity in nitric oxide signalling. J. Exp. Bot..

[3] Lindermayr C, Saalbach G, Durner J (2005). Proteomic identification of S-nitrosylated proteins in Arabidopsis. Plant Physiol..

[4] Begara-Morales JC (2015). Differential molecular response of monodehydroascorbate reductase and glutathione reductase by nitration and S-nitrosylation. J. Exp. Bot..

[5] Holtgrefe S (2008). Regulation of plant cytosolic glyceraldehyde 3-phosphate dehydrogenase isoforms by thiol modifications. Physiol. Plant..

[6] Lindermayr C, Saalbach G, Bahnweg G, Durner J (2006). Differential inhibition of Arabidopsis methionine adenosyltransferases by protein S-nitrosylation. J. Biol. Chem..

[7] Yun BW (2011). S-nitrosylation of NADPH oxidase regulates cell death in plant immunity. Nature.

[8] Chaki M (2009). Involvement of reactive nitrogen and oxygen species (RNS and ROS) in sunflower mildew interaction. Plant Cell Physiol..

[9] Begara-Morales JC (2014). Dual regulation of cytosolic ascorbate peroxidase (APX) by tyrosine nitration and S-nitrosylation. J. Exp. Bot..

[10] Hu J (2015). Site-specific nitrosoproteomic identification of endogenously S-nitrosylated proteins in Arabidopsis. Plant Physiol..

[11] Jahnová, J., Luhová, L. & Petřivalský, M. S-nitrosoglutathione reductase—the master regulator of protein S-Nitrosation in plant NO signaling. *Plants (Basel)* **8**, 48 (2019). 10.3390/plants8020048 PMC6409631 30795534

[12] Lee U, Wie C, Fernandez BO, Feelisch M, Vierling E (2008). Modulation of nitrosative stress by S-nitrosoglutathione reductase is critical for thermotolerance and plant growth in Arabidopsis. Plant Cell.

[13] Kwon E (2012). AtGSNOR1 function is required for multiple developmental programs in Arabidopsis. Planta.

[14] Xu S, Guerra D, Lee U, Vierling E (2013). S-nitrosoglutathione reductases are low-copy number, cysteine-rich proteins in plants that control multiple developmental and defense responses in Arabidopsis. Front. Plant Sci..

[15] Tichá T (2017). Characterization of S-nitrosoglutathione reductase from Brassica and Lactuca spp. and its modulation during plant development. Nitric Oxide.

[16] Gong B, Yan Y, Zhang L, Cheng F, Liu Z, Shi Q (2019). Unravelling GSNOR-mediated S-nitrosylation and multiple developmental programs in tomato plants. Plant Cell Physiol..

[17] Hussain A, Yun BW, Kim JH, Gupta KJ, Hyung NI, Loake GJ (2019). Novel and conserved functions of S-nitrosoglutathione reductase in tomato. J. Exp. Bot..

[18] Feechan A (2005). A central role for S-nitrosothiols in plant disease resistance. Proc. Natl Acad. Sci. USA.

[19] Tada Y (2008). Plant immunity requires conformational charges of NPR1 via S-nitrosylation and thioredoxins. Science.

[20] Yun BW (2016). Nitric oxide and S-nitrosoglutathione function additively during plant immunity. N. Phytol..

[21] Rusterucci C, Espunya MC, Diaz M, Chabannes M, Martinez MC (2007). S-nitrosoglutathione reductase affords protection against pathogens in Arabidopsis, both locally and systemically. Plant Physiol..

[22] Piterková J (2009). Local and systemic production of nitric oxide in tomato responses to powdery mildew infection. Mol. Plant Pathol..

[23] Tichá T (2018). Involvement of S-nitrosothiols modulation by S-nitrosoglutathione reductase in defence responses of lettuce and wild Lactuca spp. to biotrophic mildews. Planta.

[24] Jahnová J (2020). Differential modulation of S-nitrosoglutathione reductase and reactive nitrogen species in wild and cultivated tomato genotypes during development and powdery mildew infection. Plant Physiol. Biochem..

[25] Meng Y, Zhang Q, Ding W, Shan W (2014). Phytophthora parasitica: a model oomycete plant pathogen. Mycology.

[26] Nowicki M, Foolad MR, Nowakowska M, Kozik EU (2011). Potato and tomato late blight caused by *Phytophthora infestans*: an overview of pathology and resistance breeding. Plant Dis..

[27] Piterková J (2011). Dual role of nitric oxide in Solanum spp.—*Oidium neolycopersici* interactions. Environ. Exp. Bot..

[28] Kubienová L (2013). Structural and functional characterization of a plant S-nitrosoglutathione reductase from *Solanum lycopersicum*. Biochimie.

[29] Foolad MR, Merk HL, Ashrafi H (2008). Genetics, genomics and breeding of late blight and early blight resistance in tomato. CRC Crit. Rev. Plant Sci..

[30] Elsayed AY, da Silva DJH, Souza Carneiro PC, Gomide Mizubiti ES (2012). The inheritance of late blight resistance derived from Solanum habrochaites. Crop Breed. Appl. Biotechnol..

[31] Lindermayr C, Durner J (2009). S-Nitrosylation in plants: pattern and function. J. Proteom..

[32] Yu M, Yun BW, Spoel SH, Loake GJ (2012). A sleigh ride through the SNO: regulation of plant immune function by protein S-nitrosylation. Curr. Opin. plant Biol..

[33] Janus Ł (2013). Normoergic NO-dependent changes, triggered by a SAR inducer in potato, create more potent defense responses to *Phytophthora infestans*. Plant Sci..

[34] Valderrama R (2007). Nitrosative stress in plants. FEBS Lett..

[35] Jaffrey SR, Erdjument-Bromage H, Ferris CD, Tempst P, Snyder SH (2001). Protein S-nitrosylation: a physiological signal for neuronal nitric oxide. Nat. Cell Biol..

[36] Foyer CH, Noctor G (2005). Redox homeostasis and antioxidant signaling: a metabolic interface between stress perception and physiological responses. Plant Cell.

[37] Correa-Aragunde N, Foresi N, Delledonne M, Lamattina L (2013). Auxin induces redox regulation of ascorbate peroxidase 1 activity by S-nitrosylation/denitrosylation balance resulting in changes of root growth pattern in Arabidopsis. J. Exp. Bot..

[38] Yang H (2015). S-nitrosylation positively regulates ascorbate peroxidase activity during plant stress responses. Plant Physiol..

[39] Puyaubert J, Fares A, Rézé N, Peltier JB, Baudouin E (2014). Identification of endogenously S-nitrosylated proteins in Arabidopsis plantlets: Effect of cold stress on cysteine nitrosylation level. Plant Sci..

[40] Jedelská T, Kraiczová VŠ, Berčíková L, Činčalová L, Luhová L, Petřivalský M (2019). Tomato root growth inhibition by salinity and cadmium is mediated by S-nitrosative modifications of ROS metabolic enzymes controlled by S-nitrosoglutathione reductase. Biomolecules.

[41] Sels J, Mathys J, De Coninck BMA, Cammue BPA, De Bolle MFC (2008). Plant pathogenesis-related (PR) proteins: a focus on PR peptides. Plant Physiol. Biochem..

[42] Iseli B, Boller T, Neuhaus JM (1993). The N-terminal cysteine-rich domain of tobacco class I chitinase is essential for chitin binding but not for catalytic or antifungal activity. Plant Physiol..

[43] Park CJ, Seo YS (2015). Heat shock proteins: a review of the molecular chaperones for plant immunity. Plant Pathol. J..

[44] Maldonado-Alconada AM (2011). Proteomic analysis of Arabidopsis protein S-nitrosylation in response to inoculation with *Pseudomonas syringae*. Acta Physiol. Plant..

[45] Martínez-Ruiz A (2005). S-nitrosylation of Hsp90 promotes the inhibition of its ATPase and endothelial nitric oxide synthase regulatory activities. Proc. Natl Acad. Sci. USA.

[46] Huang B, Li FA, Wu CH, Wang DL (2012). The role of nitric oxide on rosuvastatin-mediated S-nitrosylation and translational proteomes in human umbilical vein endothelial cells. Proteome Sci..

[47] Pajares M (2015). Redox control of protein degradation. Redox Biol..

[48] Nakamura T (2013). Aberrant protein S-nitrosylation in neurodegenerative diseases. Neuron.

[49] Dielen AS, Badaoui S, Candresse T, German-Retana S (2010). The ubiquitin/26S proteasome system in plant-pathogen interactions: a never-ending hide-and-seek game. Mol. Plant Pathol..

[50] Bhaskar PB (2008). Sgt1, but not Rar1, is essential for the RB-mediated broad-spectrum resistance to potato late blight. BMC Plant Biol..

[51] Ballvora A (2002). The R1 gene for potato resistance to late blight (*Phytophthora infestans*) belongs to the leucine zipper/NBS/LRR class of plant resistance genes. Plant J..

[52] Qutob D, Tedman-Jones J, Gijzen M (2006). Effector-triggered immunity by the plant pathogen Phytophthora. Trends Microbiol..

[53] Du Y, Berg J, Govers F, Bouwmeester K (2015). Immune activation mediated by the late blight resistance protein R1 requires nuclear localization of R1 and the effector AVR1. N. Phytol..

[54] Sevilla F (2015). The thioredoxin/peroxiredoxin/sulfiredoxin system: current overview on its redox function in plants and regulation by reactive oxygen and nitrogen species. J. Exp. Bot..

[55] Kneeshaw S, Gelineau S, Tada Y, Loake GJ, Spoel SH (2014). Selective protein denitrosylation activity of Thioredoxin-h5 modulates plant Immunity. Mol. Cell.

[56] Romero-Puertas MC, Delledonne M (2007). S-nitrosylation of peroxiredoxin II E promotes peroxynitrite-mediated tyrosine nitration. Free Radic. Res..

[57] Camejo D (2013). Salinity-induced changes in S-nitrosylation of pea mitochondrial proteins. J. Proteom..

[58] Chang AH (2014). Respiratory substrates regulate S-nitrosylation of mitochondrial proteins through a thiol-dependent pathway. Chem. Res. Toxicol..

[59] Wang SB (2011). Redox regulation of mitochondrial ATP synthase: implications for cardiac resynchronization therapy. Circ. Res..

[60] Miedes E, Vanholme R, Boerjan W, Molina A (2014). The role of the secondary cell wall in plant resistance to pathogens. Front. Plant Sci..

[61] Böhm FMLZ, Ferrarese MDLL, Zanardo DIL, Magalhaes JR, Ferrarese-Filho O (2010). Nitric oxide affecting root growth, lignification and related enzymes in soybean seedlings. Acta Physiol. Plant..

[62] Enkhardt U, Pommer U (2000). Influence of nitric oxide and nitrite on the activity of cinnamic acid 4-hydroxylase of Zea mays in vitro. J. Appl. Bot..

[63] Monzón GC, Regente M, Pinedo M, Lamattina L, de la Canal L (2015). Effects of nitric oxide on sunflower seedlings: a balance between defense and development. Plant Signal. Behav..

[64] Jain P, von Toerne C, Lindermayr C, Bhatla SC (2018). S-nitrosylation/denitrosylation as a regulatory mechanism of salt stress sensing in sunflower seedlings. Physiol. Plant..

[65] Romero JM, Carrizo ME, Curtino JA (2018). Characterization of human triosephosphate isomerase S-nitrosylation. Nitric Oxide.

[66] Wang J (2017). Nitric oxide modifies root growth by S-nitrosylation of plastidial glyceraldehyde-3-phosphate dehydrogenase. Biochem. Biophys. Res. Commun..

[67] Swatek KN, Graham K, Agrawal GK, Thelen JJ (2011). The 14-3-3 isoforms Chi and Epsilon differentially bind client proteins from developing Arabidopsis seed. J. Proteome Res..

[68] Sedlářová M, Binarová P, Lebeda A (2001). Changes in microtubular alignment in Lactuca spp. (Asteraceae) epidermal cells during early stages of infection by *Bremia lactucae* (Peronosporaceae). Phyton.

[69] Kasprowicz A, Szuba A, Volkmann D, Baluska F, Wojtaszek P (2009). Nitric oxide modulates dynamic actin cytoskeleton and vesicle trafficking in a cell type-specific manner in root apices. J. Exp. Bot..

[70] Pasqualini S (2015). Roles for NO and ROS signalling in pollen germination and pollen-tube elongation in *Cupressus arizonica*. Biol. Plant..

[71] Rodríguez-Serrano M (2014). 2,4-Dichlorophenoxyacetic acid promotes S-nitrosylation and oxidation of actin affecting cytoskeleton and peroxisomal dynamics. J. Exp. Bot..

[72] Bradford MM (1976). Rapid and sensitive method for quantitation of microgram quantities of protein utilizing principle of protein-dye binding. Anal. Biochem..

[73] Xue Y (2010). GPS-SNO: computational prediction of protein S-nitrosylation sites with a modified GPS algorithm. PLoS ONE.

[74] Xu Y, Ding J, Wu LY, Chou KC (2013). iSNO-PseAAC: predict cysteine S-nitrosylation sites in proteins by incorporating position specific amino acid propensity into pseudo amino acid composition. PLoS ONE.

[75] Clark D, Durner J, Navarre DA, Klessig DF (2000). Nitric oxide inhibition of tobacco catalase and ascorbate peroxidase. Mol. Plant-Microbe Interact..

[76] Lin A (2012). Nitric oxide and protein S-nitrosylation are integral to hydrogen peroxide-induced leaf cell death in rice. Plant Physiol..

[77] Tanou G (2012). Oxidative and nitrosative‐based signaling and associated post‐translational modifications orchestrate the acclimation of citrus plants to salinity stress. Plant J.

[78] Correa-Aragunde N, Foresi N, Lamattina L (2015). Nitric oxide is a ubiquitous signal for maintaining redox balance in plant cells: regulation of ascorbate peroxidase as a case study. J. Exp. Bot..

[79] de Pinto MC (2013). S-nitrosylation of ascorbate peroxidase is part of programmed cell death signaling in tobacco Bright Yellow-2 cells. Plant Physiol..

[80] Fares A, Rossignol M, Peltier JB (2011). Proteomics investigation of endogenous S-nitrosylation in Arabidopsis. Biochem. Biophys. Res. Commun..

[81] Kato H, Takemoto D, Kawakita K (2013). Proteomic analysis of S‐nitrosylated proteins in potato plant. Physiol. Plant..

[82] Abat JK, Saigal P, Deswal R (2008). S-Nitrosylation—another biological switch like phosphorylation?. Physiol. Mol. Biol. Plants.

[83] Cheng T (2015). Quantitative proteomics analysis reveals that S-nitrosoglutathione reductase (GSNOR) and nitric oxide signaling enhance poplar defense against chilling stress. Planta.

[84] Tanou G, Job C, Belghazi M, Molassiotis A, Diamantidis G, Job D (2010). Proteomic signatures uncover hydrogen peroxide and nitric oxide cross-talk signaling network in citrus plants. J. Proteome Res..

[85] Eaton P (2003). Reversible cysteine-targeted oxidation of proteins during renal oxidative stress. J. Am. Soc. Nephrol..

[86] Vescovi M, Zaffagnini M, Festa M, Trost P, Schiavo FL, Costa A (2013). Nuclear accumulation of cytosolic glyceraldehyde-3-phosphate dehydrogenase in cadmium-stressed Arabidopsis roots. Plant Physiol..

[87] Wawer I (2010). Regulation of Nicotiana tabacum osmotic stress-activated protein kinase and its cellular partner GAPDH by nitric oxide in response to salinity. Biochem. J..

[88] Henry E, Fung N, Liu J, Drakakaki G, Coaker G (2015). Beyond glycolysis: GAPDHs are multi-functional enzymes involved in regulation of ROS, autophagy, and plant immune responses. PLoS Genet.

[89] Testard A (2016). Calcium-and nitric oxide-dependent nuclear accumulation of cytosolic glyceraldehyde-3-phosphate dehydrogenase in response to long chain bases in tobacco BY-2 cells. Plant Cell Physiol..

[90] Tanou G (2009). Proteomics reveals the overlapping roles of hydrogen peroxide and nitric oxide in the acclimation of citrus plants to salinity. Plant J..

[91] Bedhomme M (2012). Glutathionylation of cytosolic glyceraldehyde-3-phosphate dehydrogenase from the model plant Arabidopsis thaliana is reversed by both glutaredoxins and thioredoxins in vitro. Biochem. J..

[92] Zaffagnini M (2013). Mechanisms of nitrosylation and denitrosylation of cytoplasmic glyceraldehyde-3-phosphate dehydrogenase from Arabidopsis thaliana. J. Biol. Chem..

[93] Doulias PT (2010). Structural profiling of endogenous S-nitrosocysteine residues reveals unique features that accommodate diverse mechanisms for protein S-nitrosylation. Proc. Natl. Acad. Sci..

