# Supplementary material for: Protein S-nitrosation differentially modulates tomato responses to infection by hemi-biotrophic oomycetes of Phytophthora spp
Source: Hortic Res. 2021 Feb 1;8:34. doi: 10.1038/s41438-021-00469-3 (PMC7848004; doi:10.1038/s41438-021-00469-3)
Supplement: Supplementary file 1 — Supplementary Figures [file 41438_2021_469_MOESM1_ESM.pdf]

Supplementary Figure S1

A B

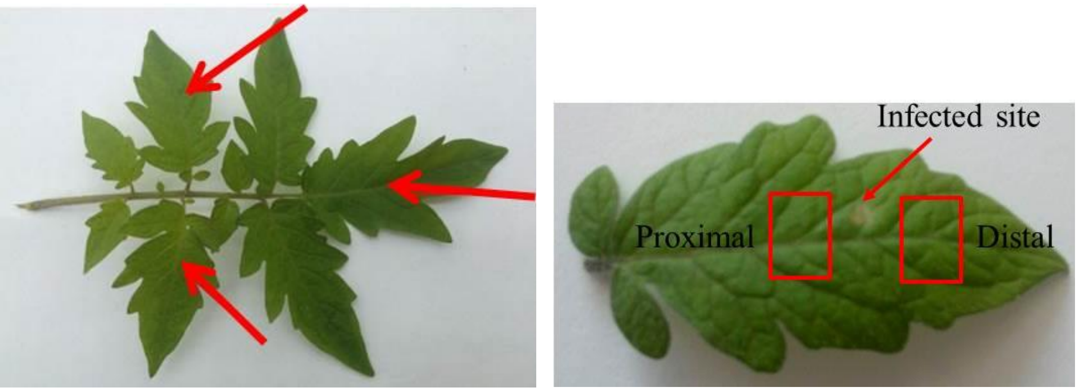

Tomato inoculation and sampling for confocal microscopy

(A) Leaves of 50 days old tomato plants were inoculated by infiltration of *Phytophthora infestans* or *P. parasitica* zoospore suspension into the leaf blade parenchyma. Three leaflets per each 4th to 7th odd pinnate leaf on a plant were inoculated, as indicated by red arrows. (B) To localize S-nitrosothiols and GSNOR within tissues, cross-sections were prepared through parts proximal as well as distal to the infected site and subjected to histochemical or immunohistochemical staining, followed by confocal microscopy imaging.

Supplementary Figure S2

A

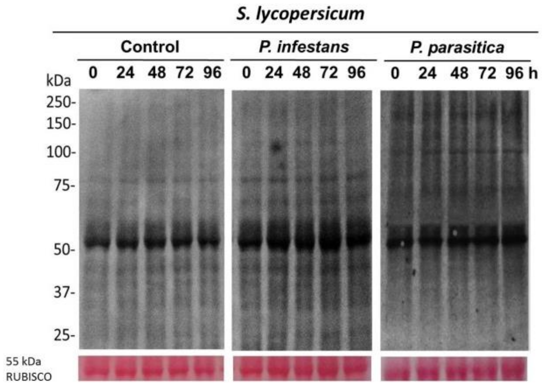

B

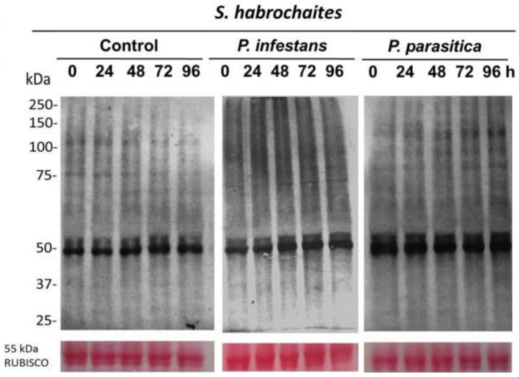

C

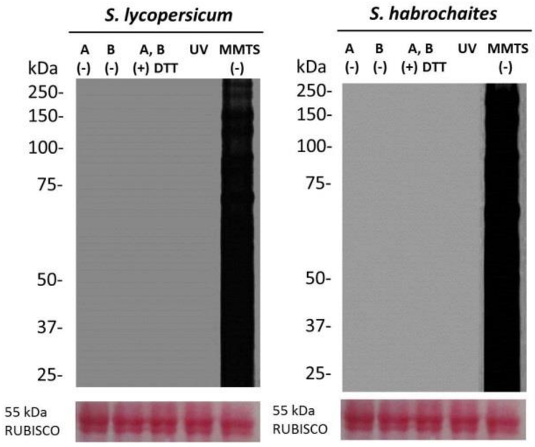

Immunodetection of S-nitrosylated proteins in tomato leaves by biotin-switch technique. Leaf samples of *S. lycopersicum* (A) and *S. habrochaites* (B) plants were collected 0, 24, 48, 72, 96 h post inoculation with *P. infestans* or *P. parasitica*. Extracted leaf proteins were subjected to the biotin switch technique (BST), purified biotinylated proteins loaded to a non-reducing SDS-PAGE gel and after an electrophoretic separation blotted onto a nitrocellulose membrane. Detection of biotinylated proteins was achieved using an anti-biotin antibody (dilution 1:10000).

(C) As negative controls, protein samples were incubated omitting 1 mM ascorbate (lanes A-) or 1 mM biotin-HPDP treatment (lanes B-) during the labelling step, or incubated without MMTS treatment during the blocking step of BST. Alternatively, S-nitrosothiols were exposed to UV light before BST (lanes UV) to decompose S-NO bond, or labelled S-nitrosylated proteins were reduced with 100 mM DTT (A, B + DTT). Numbers on the *left* of representative blot images indicate the molecular masses of protein molecular weight markers given in kDa.

Supplementary Figure S3

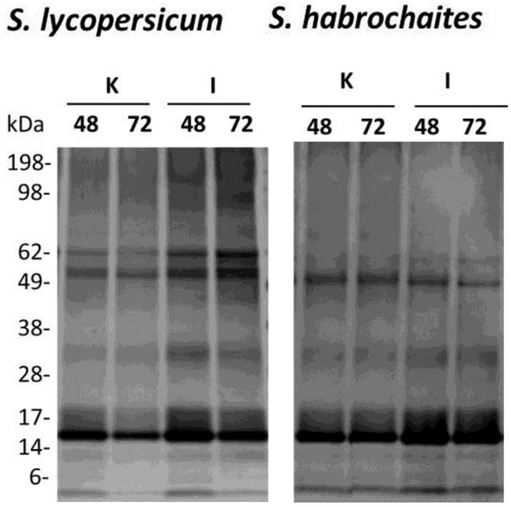

Detection of protein S-nitrosothiols during *P. parasitica* pathogenesis on *Solanum* spp. genotypes. Ten mg of proteins from tomato leaves inoculated with *P. parasitica* were subjected to the biotin switch analysis and biotinylated proteins purified on a neutravidine matrix. Eluates were separated by SDS-PAGE and proteins visualized by silver staining. The masses of protein standards are shown on the *left*.
