# Supplementary material for: Protein S-nitrosation differentially modulates tomato responses to infection by hemi-biotrophic oomycetes of Phytophthora spp
Source: Hortic Res. 2021 Feb 1;8:34. doi: 10.1038/s41438-021-00469-3 (PMC7848004; doi:10.1038/s41438-021-00469-3)
Supplement: Supplementary file 2 — Supplementary Table S1 [file 41438_2021_469_MOESM2_ESM.pdf]

Supplementary Table 1 Proteomic analysis summary for S-nitrosated proteins identified in infected tomato genotypes 72hpi

Plant species	Accession	Description	MW (kDa)	pI	Sum (Coverage)	Sum (# Proteins)	Sum (# Peptides)	Sum (# Unique Peptides)
S. lycopersicum cv. Amateur	1729860	26S protease regulatory subunit 6A homolog	48	4,94	16%	1	6	6
	6066418	Ascorbate peroxidase [Solanum lycopersicum]	27	5,73	46%	3	20	1
	66475036	Dehydroascorbate reductase [Solanum lycopersicum]	24	6,32	6%	1	2	2
	350534566	Basic 30 kDa endochitinase precursor [Solanum lycopersicum]	34	6,19	33%	1	5	1
	350534978	S-adenosylmethionine synthase 1 [Solanum lycopersicum]	43	5,51	13%	2	3	3
	350535016	Thioredoxin peroxidase 1 [Solanum lycopersicum]	17	5,18	25%	1	4	4
	350538295	Enolase [Solanum lycopersicum]	48	5,68	51%	1	16	7
	350539844	Caffeoyl-CoA O-methyltransferase [Solanum lycopersicum]	27	5,40	21%	1	4	4
	460376868	Heat shock protein 83 [Solanum lycopersicum]	81	5,01	46%	2	20	1
	460377572	14-3-3-like protein [Solanum lycopersicum]	29	4,69	3%	12	1	1
	460380444	Chaperone protein ClpB1 [Solanum lycopersicum]	101	5,82	6%	4	3	3
	460382036	Triosephosphate isomerase, cytosolic [Solanum lycopersicum]	27	5,99	63%	1	17	9
	460413941	Heat shock 70 kDa protein 15-like [Solanum lycopersicum]	93	5,22	46%	2	20	1
	460416045	ATP synthase subunit alpha, mitochondrial [Solanum lycopersicum]	55	5,50	17%	1	7	7
	544163598	ATP synthase CF1 alpha subunit (chloroplast) [Solanum lycopersicum]	55	5,14	16%	1	6	1
	806776532	Glyceraldehyde-3-phosphate dehydrogenase [Solanum lycopersicum]	37	6,34	11%	1	4	4
	816197592	Monodehydroascorbate reductase (NADH)-like protein [Solanum lycopersicum]	52	6,81	48%	2	14	14
	1052789122	Actin [Solanum lycopersicum]	42	5,31	10%	1	3	3
S. habrochaites								
	1729860	26S protease regulatory subunit 6A homolog	48	4,94	16%	1	6	6
	6066418	Ascorbate peroxidase [Solanum lycopersicum]	27	5,73	4%	3	1	1
	350539844	Caffeoyl-CoA O-methyltransferase [Solanum lycopersicum]	27	5,40	21%	1	4	4
	460382243	Glyceraldehyde-3-phosphate dehydrogenase A, chloroplastic [Solanum lycopersicum]	43	8,46	23%	1	7	7
	460408278	Enolase-like [Solanum lycopersicum]	48	5,99	57%	1	20	8
	460413941	Heat shock 70 kDa protein 15-like [Solanum lycopersicum]	93	5,22	46%	2	20	1
	544163598	ATP synthase CF1 alpha subunit (chloroplast) [Solanum lycopersicum]	55	5,14	16%	1	6	1
	723713146	Putative late blight resistance protein homolog R1B-16 [Solanum lycopersicum]	103	8,44	11%	1	9	9
	823683802	S-adenosylmethionine synthase 2 [Solanum lycopersicum]	43	5,41	13%	2	3	3
	957571263	Actin-7 [Solanum lycopersicum]	42	5,31	17%	1	5	5
	1018443168	Actin [Solanum lycopersicum]	42	5,30	10%	1	3	3
